# State-Aware RNA Biomarkers in Triple-Negative Breast Cancer (TNBC): Integrating Tumor Plasticity, Spatial Architecture, and Temporal Monitoring

**DOI:** 10.3390/ijms27114692

**Published:** 2026-05-22

**Authors:** Amal Qattan

**Affiliations:** 1 Innovation and Research Division, King Faisal Specialist Hospital and Research Centre, Riyadh 11211, Saudi Arabia; akattan@kfshrc.edu.sa; 2 College of Medicine, Alfaisal University, Riyadh 11533, Saudi Arabia

**Keywords:** triple-negative breast cancer (TNBC), RNA biomarkers, non-coding RNA, state-aware biomarkers, tumor plasticity, transcriptional reprogramming, tumor microenvironment, spatial transcriptomics, liquid biopsy, circulating tumor RNA, therapy resistance, adaptive oncology, cell state transitions, precision oncology, dynamic biomarkers

## Abstract

Triple-negative breast cancer is defined by the absence of druggable receptor targets and by a biologically dynamic phenotype that renders static, single-timepoint biomarker strategies fundamentally inadequate. Current predictive markers, including PD-L1 expression, tumor mutational burden, and genomic profiling, fail to capture the therapy-induced transcriptional reprogramming, spatial heterogeneity, and drug-tolerant persister states that drive resistance and relapse. In this review, we argue that RNA, particularly non-coding RNA (ncRNA), represents a complementary and state-aware platform for biomarker development in TNBC, capable of capturing transcriptional adaptation, regulatory threshold dynamics, and cell state transitions that static genomic markers cannot fully detect. Unlike messenger RNAs, which reflect active transcriptional programs, long non-coding RNAs and circular RNAs modulate the stability of state transitions and are specifically induced under conditions of therapeutic stress, immune exclusion, and drug tolerance, which are properties that make them suitable as potential early and sensitive indicators of adaptive reprogramming. We review the biological rationale for RNA as a state-aware readout across five dimensions: tumor plasticity, immune context, stress response, therapy adaptation, and microenvironment composition. An examination is conducted regarding how spatial transcriptomics can map RNA-defined resistant niches within TNBC, how serial liquid biopsy RNA measurements, including extracellular vesicle RNA and circulating tumor RNA, enable temporal monitoring of transcriptional state shifts before radiologic progression, and what analytical and clinical standards deployable RNA assays must meet. Finally, a state-guided adaptive management framework is proposed in which RNA signatures function as iteratively updated measurement layers informing therapy selection, on-treatment monitoring, and early resistance detection. This review outlines trial design models and defines the validation standards required before RNA-guided adaptation can enter clinical practice.

## 1. Introduction

Triple-negative breast cancer (TNBC) is one of the most clinically intractable subtypes of breast cancer, defined by the absence of estrogen receptor, progesterone receptor, and HER2 expression [1]. It is characterized by aggressive biology, high rates of early metastasis, and limited targeted therapeutic options [1]. Despite incremental advances in immunotherapy and antibody–drug conjugates (ADCs), the majority of patients with advanced TNBC still face poor long-term outcomes, and the field continues to struggle with the absence of reliable, actionable biomarkers that can guide therapy selection and monitoring in real time [2]. The central challenge is not a lack of candidate biomarkers, but a fundamental mismatch between the measurement strategies currently deployed and the biology of the disease being measured.

TNBC is not a fixed entity but a biologically dynamic system whose transcriptional programs shift under selective pressure, therapeutic stress, and microenvironmental influence [3]. Unlike genomic alterations, which define lineage and mutational burden, RNA signatures capture dynamic tumor states and therapy-induced adaptation, offering a potentially complementary and state-aware layer for clinically actionable biomarker development in TNBC. Here, state awareness is defined as having the capacity or potential to inform the identification of and/or transitions between tumor states. In this review, we define the failure of current static biomarker strategies, present the biological rationale for RNA, particularly non-coding RNA (ncRNA), as an inherently state-aware readout, and propose a framework for clinical deployment that integrates spatial, temporal, and resistance heterogeneity. By doing so, we articulate a translational roadmap in which RNA-based signatures serve as adaptive measurement layers aligned with the dynamic biology of TNBC.

The central challenge in TNBC biomarker development is not the absence of candidate molecules, but the absence of measurement systems capable of capturing dynamic tumor state transitions. RNA-based signatures provide a state-aware measurement layer that integrates transcriptional plasticity, spatial heterogeneity, and therapy-induced adaptation, enabling biologically informed and adaptive clinical decision making. We propose that biomarkers should be designed as state-aware measurement systems rather than static molecular classifiers. Accordingly, this review differs from the prior ncRNA literature in three key aspects: (1) it frames biomarkers as dynamic measurement systems; (2) it introduces a tri-layer RNA architecture linking genomic potential, transcriptional state, and transition probability; and (3) it proposes a clinically actionable adaptive framework for RNA-guided disease monitoring and treatment modification.

## 2. The Failure of Static Biomarkers in TNBC

The clinical management of TNBC has historically been constrained by the absence of the hormone receptor and HER2 targets that define treatment selection in other breast cancer subtypes, which has left cytotoxic chemotherapy as the backbone of care for decades [1]. Although the emergence of immune checkpoint inhibitors and ADCs has expanded therapeutic options, it has simultaneously exposed a more fundamental limitation: the biomarker paradigms that guide patient selection remain largely static, while the disease they attempt to measure is intrinsically dynamic.

The central limitation of current biomarker strategies in TNBC is architectural rather than molecular. Most approved or investigational markers are derived from single-site, single-timepoint measurements applied to a malignancy defined by transcriptional plasticity, adaptive reprogramming, and microenvironmental responsiveness. While immune composition, drug sensitivity, epithelial–mesenchymal balance, and stress-response programs fluctuate under therapeutic pressure and stromal influence, clinical decision making continues to rely on assays performed on archival tissue, often obtained prior to systemic treatment. This structural mismatch between measurement design and tumor biology underlies the inconsistent predictive performance observed across PD-L1 expression, tumor mutational burden (TMB), ADC response markers, and circulating tumor DNA (ctDNA)-based minimal residual disease (MRD) approaches. Therefore, failure is not the absence of candidate biomarkers but the absence of state-aware measurement frameworks.

### 2.1. PD-L1 and Mutational Burden as Imperfect Predictors

PD-L1 expression, assessed through immunohistochemistry on diagnostic tissue, serves as the current companion diagnostic for pembrolizumab in TNBC; however, its predictive performance remains inconsistent across trials and clinical settings. Variability in assay platforms, scoring systems, and cutoffs contributes to technical heterogeneity, but biological heterogeneity is equally consequential. PD-L1 expression is spatially variable across tumor regions, differs between primary and metastatic sites, and can shift substantially under chemotherapy and immune modulation [4,5]. Emerging evidence further suggests that response to checkpoint inhibition in metastatic TNBC may depend more on immune architecture, including myeloid composition and stromal organization, than on PD-L1 ligand expression alone [6]. A single archival biopsy, therefore, cannot reliably represent the evolving tumor–immune interface that ultimately determines therapeutic response.

Tumor mutational burden (TMB), proposed as a broader genomic correlate of immunogenicity, has similarly demonstrated limited predictive utility in TNBC. While conceptually appealing, TMB reflects cumulative mutational load rather than current immune engagement, and its clinical relevance in TNBC remains inconsistent and poorly standardized [7]. Both PD-L1 and TMB illustrate a common structural limitation: static genomic or protein-level metrics are used to predict outcomes in a system whose immune context is temporally and spatially fluid.

### 2.2. Unpredictability of ADC Resistance

The unpredictability of resistance to antibody–drug conjugates (ADCs) represents a related but distinct challenge. Although agents such as sacituzumab govitecan have demonstrated meaningful activity in TNBC, resistance frequently emerges without clear genomic predictors identifiable at baseline. Mechanisms of acquired resistance appear to involve dynamic and context-dependent transcriptional adaptation, altered intracellular trafficking, drug efflux modulation, and shifts in survival signaling pathways [8]. Pre-treatment genomic or protein expression profiling has not consistently identified patients at risk of early progression, underscoring the limitations of relying on static baseline markers to anticipate adaptive therapeutic escape.

### 2.3. Genomics and MRD: Gaps in Dynamic Surveillance

While genomic profiling provides valuable information regarding lineage, clonal architecture, and evolutionary history, it does not necessarily reflect the tumor’s current functional state. Transcriptional programs governing epithelial–mesenchymal transition (EMT), stemness, immune evasion, and drug tolerance can shift rapidly and reversibly in response to therapy without corresponding changes in underlying DNA sequence [9]. Subtype switching observed during and after neoadjuvant therapy further illustrates that baseline genomic classification cannot fully predict post-treatment tumor behavior [3].

Minimal residual disease detection using circulating tumor DNA (ctDNA) represents an important advance in dynamic monitoring; however, even here limitations remain. While ctDNA positivity is associated with relapse risk in TNBC, sensitivity varies across platforms, and detection in low-burden disease remains inconsistent [10,11]. Moreover, single-timepoint assessment may miss evolving resistance clones or adaptive transcriptional states that precede detectable genomic relapse. Although longitudinal ctDNA monitoring improves risk stratification, it primarily tracks genomic alterations rather than functional reprogramming.

**Figure 1:**
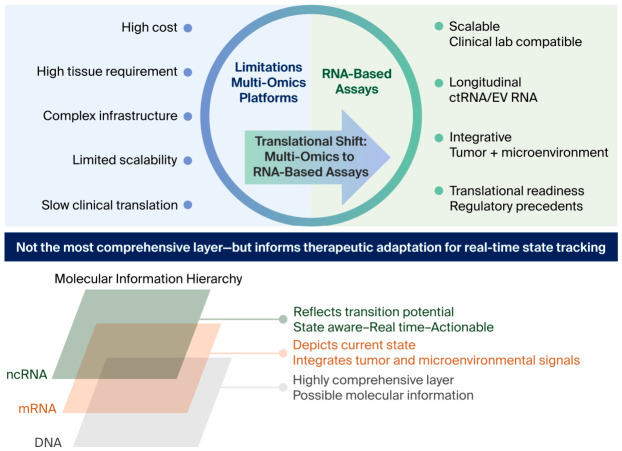
Translational positioning of RNA-based assays as clinically deployable platforms for real-time state tracking in TNBC. This figure contrasts limitations of multi-omics platforms with the clinical advantages of RNA-based assays, including scalability, laboratory compatibility, and longitudinal monitoring via ctRNA and EV RNA. The lower panel presents a molecular hierarchy in which DNA encodes potential alterations, mRNA reflects the current state, and ncRNA captures regulatory dynamics and transition potential, positioning RNA as a state-aware and clinically actionable biomarker layer.

Collectively, these examples reveal a recurring pattern: clinically relevant tumor properties in TNBC are emergent and state-dependent, yet measurement strategies remain static and reductionist. Single biopsies and single-timepoint assays capture only a fragment of the biological information required to understand where a tumor is positioned along its adaptive trajectory. Therefore, TNBC biomarker limitations arise not from a lack of molecular insight but from a reliance on measurement systems that are insufficiently responsive to dynamic tumor biology. Addressing this mismatch requires biomarker frameworks capable of capturing transcriptional architecture, spatial heterogeneity, and temporal adaptation, which are properties that align more closely with state-aware measurement. These limitations collectively indicate that the central challenge in TNBC biomarker development is not the absence of candidate molecules but the absence of measurement systems capable of capturing dynamic tumor state transitions. This is an informational layer that RNA biology is particularly well positioned to provide. Table 1 summarizes the limitations of static measures and the evidence supporting a shift toward dynamic measures. These limitations collectively support a translational shift toward measurement layers that are dynamic, scalable, and clinically deployable. The positioning of RNA-based assays within this shift is illustrated in Figure 1.

## 3. RNA as a State-Aware Readout

### 3.1. Distinguishing mRNA and ncRNA Layers in State Awareness

The transcriptome is not a static output but a dynamic regulatory architecture layered across distinct levels of biological control. Genomic alterations define lineage and evolutionary constraint, establishing the range of phenotypic possibilities available to a tumor. Messenger RNA (mRNA) profiles, in turn, describe the tumor’s instantaneous transcriptional state, reporting which pathways are currently active and which phenotypes are being expressed. However, mRNAs largely represent the outputs of regulatory decisions rather than the mechanisms that determine whether those decisions will shift.

Non-coding RNAs (ncRNAs), including long non-coding RNAs (lncRNAs), circular RNAs (circRNAs), and microRNAs (miRNAs), occupy a distinct regulatory layer that governs the stability and directionality of cell state transitions. Through modulation of chromatin architecture, post-transcriptional regulation, and signaling network topology, ncRNAs influence the thresholds at which phenotypic programs are activated, maintained, or reversed [12]. Rather than merely reflecting cellular identity, they participate in shaping the probability that a tumor will transition into alternative states under stress or therapeutic pressure.

This distinction defines a tri-layer state architecture: genomic alterations determine evolutionary potential, mRNA expression profiles describe the tumor’s current phenotype, and ncRNA networks regulate transition probability between phenotypic states. In this framework, ncRNAs function as modulators of state-transition thresholds, influencing whether adaptive programs such as epithelial–mesenchymal transition, immune exclusion, stemness, or drug tolerance are initiated and stabilized. While ncRNAs do not act independently of broader regulatory systems, their position within network control nodes renders them particularly sensitive indicators of impending state shifts.

**Figure 2:**
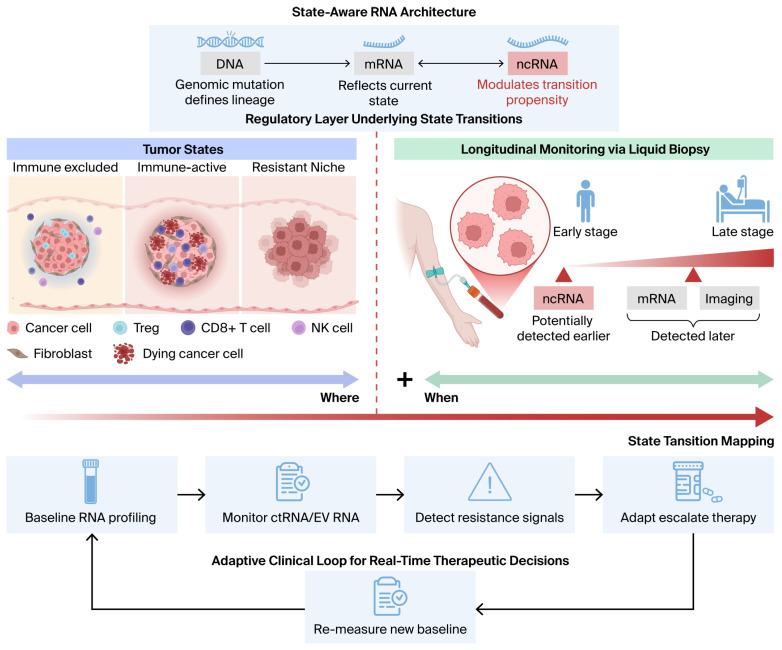
State-aware RNA architecture integrating spatial heterogeneity, temporal monitoring, and transition propensity with adaptive clinical decision making in TNBC. This figure outlines a state-aware RNA framework linking molecular regulation with tumor evolution where spatial tumor states and temporal dynamics are integrated through longitudinal liquid biopsy, and ncRNA signals may precede mRNA and imaging changes. The bottom panel represents an adaptive clinical loop enabling real-time tracking of tumor state transitions.

The biomarker implications are consequential: if genomic profiling reveals what a tumor can become and mRNA profiling reveals what it is doing at a given moment, ncRNA dynamics may reveal what it is likely to become next. This probabilistic, transition-oriented perspective positions ncRNAs not simply as additional transcriptomic features but as components of a state-aware measurement layer aligned with the adaptive biology of TNBC. This tri-layer state-aware architecture, together with its spatial and temporal extensions and clinical implications, is summarized in Figure 2.

### 3.2. ncRNAs Mark Plasticity and Stress Response

Tumor plasticity in TNBC is driven in substantial part by the regulatory architecture that ncRNAs help maintain. EMT represents a dynamic cell state transition central to TNBC plasticity, invasion, and therapeutic resistance. Within this framework, lncRNAs and circRNAs function as modulators of transition thresholds, influencing the probability and stability of shifts between epithelial and mesenchymal states rather than acting as simple regulators of static phenotypes [13]. As key regulators of epithelial–mesenchymal plasticity and cancer stemness, many lncRNAs function not as simple on/off switches but as modulators of transition thresholds that determine the propensity of a cell to shift between epithelial and mesenchymal states [9]. This threshold-modulating function is what makes ncRNAs valuable as state-aware markers, as they may influence transition propensity toward resistant states before the transition is manifested at the mRNA or protein level. It is important to take this into consideration, as ncRNAs may influence the probability and trajectory of state transitions in conjunction with broader regulatory networks. RNA represents a pragmatically accessible and biologically informative layer, rather than an exclusive solution.

Therapy-induced reprogramming represents a critical domain in which ncRNA biology is directly relevant to clinical biomarker development. Chemotherapy and targeted therapies do not simply kill sensitive cells; they also exert selective pressure that promotes transcriptional adaptation in surviving cells. Chemotherapy-mediated lncRNA expression changes can influence immune cell plasticity in the tumor microenvironment, with implications for immune evasion during and after treatment [14]. LncRNAs influence chemotherapy resistance in breast cancer by multiple mechanisms through which lncRNA expression changes under treatment contribute to acquired resistance, including modulation of drug efflux, DNA damage response, and survival signaling [15]. Importantly, these lncRNA shifts often precede the emergence of resistance at the phenotypic level, supporting their potential utility as early warning biomarkers.

### 3.3. Evidence for ncRNAs as State-Aware Markers in TNBC

Evidence supporting ncRNAs as state-aware biomarkers in TNBC emerges most clearly when organized around the biological states they encode rather than individual transcripts in isolation. Across immune regulation, stress adaptation, and drug-tolerant persistence, ncRNAs repeatedly appear at regulatory nodes that govern transition stability rather than merely reflecting downstream transcriptional outputs.

#### 3.3.1. ncRNAs and Immune Exclusion States

Immune exclusion represents a clinically decisive state in TNBC, influencing responsiveness to checkpoint inhibition and shaping therapeutic trajectories. Multiple lncRNAs have been implicated in regulating immune checkpoint expression, antigen presentation, and stromal–immune crosstalk [16]. Rather than functioning solely as passive correlates, these ncRNAs participate in regulatory programs that define whether tumors adopt immune-inflamed or immune-excluded phenotypes. For example, LINC01614 has been associated with poor prognosis in breast cancer and is regulated by TGF-β and focal adhesion kinase signaling, which are strongly implicated in stromal remodeling and immune exclusion [17]. Similarly, Li, Hu, and colleagues developed an immune-related lncRNA pair model in TNBC in which USP30-AS1 correlated with PD-L1 expression and immune response, demonstrating that lncRNA expression patterns can quantify tumor–immune interaction states with clinical relevance [18]. These findings suggest that ncRNAs may encode immune architecture and transition dynamics more sensitively than single-gene protein markers.

#### 3.3.2. ncRNAs and Drug-Tolerant Persister Programs

Drug-tolerant persister (DTP) cells represent a transient, non-genetic adaptive state that enables tumor survival under acute therapeutic stress and provides a reservoir for acquired resistance [19]. The characterization of DTP cells in TNBC has identified shared persistence programs across treatments and patients, distinguished by transcriptional features that separate persisters from bulk tumor populations [19]. Several lncRNAs implicated in transitions to states such as chromatin remodeling, stress adaptation, and epithelial–mesenchymal plasticity, including MALAT1, HOTAIR, and NEAT1, have been associated with resistance-associated transcriptional programs [20]. These ncRNAs do not independently determine resistance but are positioned within regulatory networks that influence the stability and reversibility of persister states. Their expression patterns may therefore serve as early indicators of transition into drug-tolerant phenotypes before overt clinical progression becomes evident.

#### 3.3.3. ncRNAs and Stress-Induced Plasticity

Tumor plasticity in TNBC is closely linked to the ncRNA-mediated regulation of EMT and stemness programs. Many lncRNAs and circRNAs act as modulators of EMT thresholds rather than binary switches [9,13]. By influencing chromatin accessibility, post-transcriptional regulation, and signaling network topology, ncRNAs can alter the propensity of tumor cells to shift between epithelial and mesenchymal states. Importantly, numerous ncRNAs display low baseline expression under homeostatic conditions but are highly inducible under cellular stress, including chemotherapy exposure, hypoxia, and nutrient deprivation [21]. This inducibility is biomarker-relevant: low baseline levels reduce background noise, while stress-triggered upregulation provides a temporally aligned signal during clinically meaningful transitions. Chemotherapy-mediated lncRNA shifts have been shown to influence immune plasticity and survival signaling pathways, further supporting their role in encoding adaptive reprogramming [14,15].

### 3.4. RNA Reflects Integrated Biological Dimensions

The biological rationale for ncRNAs as state-aware readouts can be summarized across five dimensions that are directly relevant to TNBC clinical biology: First, they reflect state plasticity by modulating the regulatory thresholds that determine cell state stability. Second, they encode immune context, the presence or absence of immune exclusion programs, immune checkpoint regulation, and stromal–immune crosstalk, with a specificity that single-gene mRNA markers do not capture [22]. Third, they are responsive to cellular stress, with many circRNAs and lncRNAs specifically induced under conditions of drug exposure, hypoxia, or nutrient deprivation [21]. Fourth, they capture therapy adaptation at the transcriptional level, including the emergence of alternative survival programs and the repression of drug sensitivity pathways. Fifth, lncRNAs in particular show high tissue and cell-type specificity and context dependence, making them more informative about tumor microenvironment composition than broadly expressed mRNAs [23]. Together, these properties define the “state-aware” model: while genomic mutations define lineage and mRNA reflects the current transcriptional state, ncRNAs regulate network stability and transition thresholds, and therefore may determine whether a tumor transitions into a resistant state before mRNAs reflect that state. Together, these dimensions define a state-aware measurement architecture that directly addresses the structural limitations outlined in Section 2.

### 3.5. Clinical Evidence

Clinical research into ncRNAs in TNBC has increasingly demonstrated their potential as prognostic, predictive, and state-aware biomarkers capable of capturing tumor aggressiveness, metastatic potential, and therapy adaptation. Early transcriptomic investigations established that integrated miRNA and mRNA signatures could stratify TNBC according to survival outcomes, suggesting that ncRNA profiling provides clinically relevant information beyond conventional clinicopathologic classification [24]. Similarly, deep sequencing studies identified miRNA expression patterns associated with invasiveness and prognosis in breast cancer, including signatures linked to aggressive tumor phenotypes and metastatic behavior [25]. Among the most extensively studied examples, the miR-200 family emerged as a central regulator of EMT, with loss of miR-200 expression associated with increased cellular plasticity, migration, and metastatic competence through the derepression of ZEB1 and ZEB2 [26,27]. These findings established an important conceptual framework in which ncRNA expression reflects dynamic cell state transitions rather than static molecular subtype alone. Additional studies identified circANKS1B as a promoter of metastatic progression in breast cancer through EMT-associated signaling pathways, further supporting the relevance of ncRNAs as markers of invasive and adaptive tumor states [28]. LncRNAs including HOTAIR and MALAT1 have similarly been associated with metastasis and poor prognosis, with HOTAIR linked to chromatin-state reprogramming and metastatic competence [29,30], while MALAT1 has been implicated in tumor differentiation, metastatic regulation, and transcriptional adaptation [31,32].

More recent studies have increasingly connected ncRNA biology to therapy resistance, stemness, and adaptive survival programs in TNBC. NEAT1 has been shown to promote chemoresistance and cancer stemness in TNBC, suggesting that stress-responsive lncRNAs may identify tumors transitioning toward drug-tolerant persister-like states [33]. Earlier work demonstrated that the hypoxia-induced activation of NEAT1 supports cancer cell survival, linking ncRNA expression directly to microenvironmental stress adaptation. Likewise, LINC00511 has been associated with breast cancer stemness and tumorigenesis through the regulation of pluripotency-associated pathways, supporting its potential role as a marker of stem-like and therapy-adaptive tumor states [34]. Multiple miRNAs implicated in proliferation and resistance pathways have also shown translational relevance. miR-21, one of the earliest oncogenic miRNAs identified in breast cancer, was associated with anti-apoptotic signaling and deregulated expression in malignant tissues [35,36], while the miR-34 family was linked to apoptosis regulation, stemness suppression, and metastatic inhibition [37,38]. In contrast, miR-221/222 expression has been associated with treatment resistance and aggressive tumor behavior through the regulation of cell-cycle control pathways [39]. Collectively, these studies support the notion of the clinical application of ncRNAs not merely as static biomarkers but as dynamic indicators of EMT plasticity, stress adaptation, stemness, and therapy-responsive tumor evolution in TNBC. A summary of specific ncRNAs identified as having potential utility as biomarkers with state-aware relevance is provided in Table 2.

Single-cell transcriptomics and spatial multi-omics platforms have substantially advanced the mechanistic understanding of TNBC biology. However, its translational trajectory toward routine clinical deployment requires progress regarding cost, tissue requirements, computational infrastructure, and standardized analytical pipelines applicable across sites [41,42]. It is reasonable to hypothesize that RNA-based assays can occupy a distinct and complementary translational niche. They are (1) scalable across clinical laboratory settings using established extraction and quantification platforms; (2) measurable longitudinally from minimally invasive liquid biopsy matrices, including plasma extracellular vesicles and circulating tumor RNA [43,44]; and (3) capable of simultaneously integrating signals from the tumor cell compartment and the surrounding microenvironment within a single assay readout [18,45]. Critically, targeted RNA panels, whether tissue-based or liquid biopsy-derived, have regulatory and analytical precedents that position them for clinical validation studies on timescales that spatial proteomics and full multi-omic platforms cannot match [46,47].

This scalability, longitudinal accessibility, microenvironmental integrative capacity, and relative proximity to clinical deployment may collectively define RNA not as a reductive alternative to richer “omic” modalities but as the most pragmatically actionable layer for state-aware biomarker strategies in TNBC at this stage of translational readiness. While multi-omic platforms provide broader molecular resolution, their complexity can limit clinical scalability, whereas RNA-based assays may offer a uniquely deployable interface for real-time monitoring of tumor state. In practical terms, this framework suggests that genomic data define possibility, mRNA defines current state, and ncRNA defines transition potential.

RNA biomarkers are most appropriately positioned as a complementary layer within a multi-modal monitoring framework rather than as replacements for established tools. CtDNA tracks clonal genomic evolution and stratifies relapse risk in TNBC [11,48]; however, its sensitivity in low-burden disease remains limited. It should be noted that studies quantitatively comparing the sensitivity, specificity, or lead time of RNA markers with ctDNA are currently lacking. However, EV RNA and circulating tumor RNA may detect transcriptional reprogramming in precisely these windows where ctDNA signal is absent, and there is potential for RNA-ctDNA co-measurement from the same plasma draw to improve MRD resolution [47]. Imaging captures radiologic progression as a late downstream consequence of biological changes that RNA signatures may reflect earlier, making RNA-triggered clinical reassessment a logical potential bridge between ctDNA surveillance and radiologic confirmation. Spatial transcriptomics may complement systemic liquid biopsy signals by localizing resistance programs to specific tumor niches, informing whether resistance is diffuse or spatially restricted [49,50], while RNA-based immune state profiling may add temporal resolution to immune monitoring that static PD-L1 immunohistochemistry cannot provide [5,6]. It may also be possible, however, to apply longitudinal analysis to protein markers such as PD-L1. The integrative logic follows the detection hierarchy proposed in this review: ncRNA shifts earliest, mRNA consolidates the transcriptional state, ctDNA captures clonal expansion, and imaging documents phenotypic consequence, with each modality occupying a distinct and non-redundant position along the tumor’s adaptive trajectory.

## 4. Spatially Informed RNA Signatures

While regulatory RNA layers define transition potential, understanding where these transitions occur requires spatial resolution. The recognition that TNBC is not a uniform transcriptional entity but a spatially organized mosaic of distinct cell states has been substantially advanced by the adoption of spatial transcriptomics technologies. Spatial transcriptomics enables the measurement of gene expression at defined positions within a tissue section, allowing RNA-defined cell states to be mapped onto tumor architecture and correlated with clinically relevant features, such as proximity to the invasive margin, immune infiltrate distribution, and stromal composition [51]. The application of this technology to TNBC has begun to reveal the architecture of resistant niches, spatially restricted regions within a tumor that harbor transcriptional programs associated with therapy evasion, and to connect RNA expression patterns to ADC resistance, immune exclusion, and persister biology in ways that single-cell or bulk analyses cannot.

Wang et al. applied spatial transcriptomics to a multi-cohort TNBC dataset and documented substantial intratumoral heterogeneity in transcriptional state, with distinct spatial domains corresponding to immune-active, immune-excluded, and stromal-enriched niches [49]. A conserved spatial architecture has been demonstrated in TNBC, characterized by compartmentalized regions of immune activity and immune exclusion, with RNA-defined boundaries between these compartments that were spatially consistent across a diverse patient cohort [52]. These findings suggest that the spatial organization of RNA-defined states is not random but follows a structured program that may have predictive implications for therapy response. Molecular landscapes revealed by spatial transcriptomics in TNBC show spatial patterns of immune gene expression and provide substantially more information about immunotherapy responsiveness than bulk tumor measurements [41].

The connection between spatially resolved RNA signatures and ADC resistance is an area of active investigation. Spatial transcriptomics applied to breast cancer samples demonstrated that drug responses vary by spatial tumor microenvironment context, with distinct transcriptional niches showing differential sensitivity to targeted therapies [50]. A similar methodology exploring metabolic pathway heterogeneity in TNBC immunotherapy found that the spatial co-localization of metabolic and immune transcriptional programs within the tumor microenvironment predicted treatment outcomes more accurately than bulk expression analysis [53]. These spatial analyses suggest that resistance to ADCs may be concentrated in specific niches with distinct RNA expression profiles, rather than being a global property of the tumor.

ncRNA gradients across tumor margins represent an emerging dimension of spatially informed biomarker development. Spatial RNA biology can be explored in archival formalin-fixed paraffin-embedded (FFPE) tissues, opening the possibility of retrospective spatial lncRNA analysis in clinically annotated cohorts [54]. Spatial transcriptomics to identify prognostic lncRNAs in colorectal cancer revealed that the spatial distribution of lncRNA expression across tumor regions was more prognostically informative than their average expression levels, a principle likely applicable to TNBC [55]. Spatial analysis of the TNBC tumor microenvironment in patients with residual disease after neoadjuvant chemo-immunotherapy identified distinct spatial RNA programs in areas of persisting tumors that differed from treatment-naive tissue [56]. The spatial distribution of lncRNAs, the non-coding transcript component of the spatial transcriptome, remains a relatively underexplored dimension. However, Xu et al. noted that lncRNA expression in tissue reveals specificity and functional roles in cancer that cannot be determined through single-cell or bulk analyses [57]. RNA-based spatial profiling captures functional states of both the tumor and microenvironment simultaneously, whereas protein or genomic markers often isolate single pathways without integrating context.

## 5. Temporal RNA Biomarkers

One of the most compelling properties of RNA as a potential clinical biomarker is its measurability in liquid biopsy format across serial timepoints, enabling the longitudinal tracking of tumor state changes without repeated tissue biopsies. Circulating tumor RNA (ctRNA), RNA carried within extracellular vesicles (EV RNA), and exosomal non-coding RNAs may provide windows into the dynamic transcriptional programs of the tumor and its microenvironment in real time. The alignment of this capability with clinical needs in TNBC, MRD detection, therapy monitoring, early detection of resistance, and adaptive treatment decisions makes temporal RNA biomarkers a priority for translational development.

What is measurable today in clinical and research settings includes ctDNA-based MRD approaches that are increasingly validated, EV-based RNA profiling from plasma, and multi-miRNA panels from tumor-derived extracellular vesicles. Kim et al. (2021) demonstrated that a multi-miRNA panel derived from tumor extracellular vesicles could serve as a diagnostic biomarker for early-stage breast cancer, with sensitivity and specificity exceeding those of individual miRNAs [58]. Sadovska et al. (2022) performed a comprehensive characterization of RNA cargo in extracellular vesicles from breast cancer patients undergoing neoadjuvant chemotherapy, documenting that EV RNA profiles change substantially during treatment and that these changes reflect transcriptional adaptations occurring in the tumor [43]. Su et al. (2021) showed that plasma EV long RNA profiles could differentiate breast cancer patients from healthy controls and predict treatment response, providing proof of principle for the clinical utility of this approach [44].

What remains experimental includes spatial–temporal integration, the combination of serial liquid biopsy RNA data with spatially resolved tissue profiling to track how resistant niches evolve during therapy. The measurement of lncRNA and circRNA species in extracellular vesicles as markers of specific transcriptional programs, such as persister cell emergence or EMT activation, is an active area of research that has not yet yielded validated clinical assays. Fang et al. (2024) reviewed the potential of EV circRNAs in breast cancer, noting that circRNA cargo in extracellular vesicles is enriched and biologically active, but that standardized methods for EV circRNA quantification from plasma samples are not yet established [59]. Zayakin et al. (2023) systematically reviewed extracellular vesicles as a source of RNA biomarkers in breast cancer liquid biopsy and concluded that EV RNA profiling can detect breast cancer with high accuracy, while the challenge of distinguishing cancer-derived from non-cancer-derived EVs in heterogeneous blood plasma is an inherent methodological difficulty [60].

**Figure 3:**
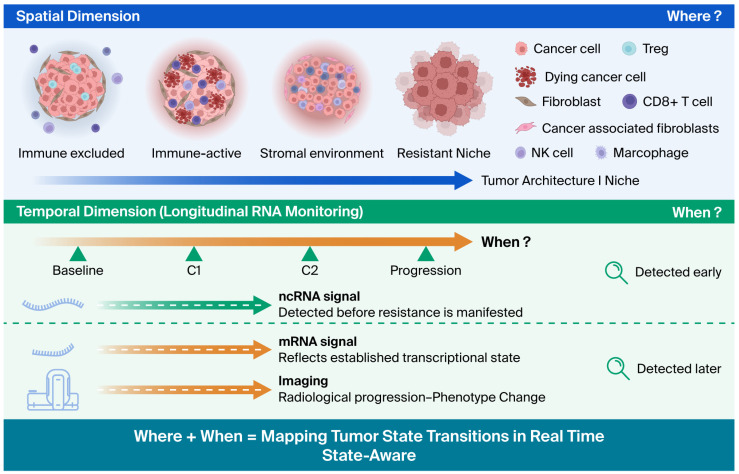
Integration of spatial and temporal RNA dimensions for real-time mapping of tumor state transitions. This figure integrates spatial (“where”) and temporal (“when”) dimensions of tumor biology within a state-aware RNA framework. Distinct tumor microenvironment architectures are linked to longitudinal RNA monitoring across disease progression. ncRNA signals emerge earlier, reflecting regulatory shifts, whereas mRNA and imaging capture later stages. The convergence of spatial context and temporal dynamics enables real-time mapping of tumor state transitions and supports RNA-based adaptive monitoring strategies for guiding therapeutic decisions.

What would define clinical utility for temporal RNA biomarkers is the demonstration that RNA program shifts detected in serial liquid biopsy samples predict treatment outcomes in advance of radiologic progression or clinical deterioration. Brogna et al. (2026) reviewed liquid biopsy approaches in TNBC and articulated criteria for clinical translation, including reproducibility, validated endpoints, and association with actionable clinical decisions [46]. The question of whether RNA shifts can predict relapse before radiology is biologically plausible: transcriptional reprogramming precedes phenotypic resistance but requires prospective validation studies with defined timepoints and outcome endpoints. Simancas-Racines et al. (2025) reviewed multi-omic liquid biopsy approaches in breast cancer and emphasized that the integration of RNA-based markers with ctDNA surveillance could improve MRD sensitivity, particularly in cases where the ctDNA signal is below detection threshold but transcriptional adaptation is already underway [47]. Rajan et al. (2025) demonstrated that the characterization of the salivary RNA landscape could identify potential diagnostic, prognostic, and follow-up biomarkers for breast cancer, suggesting that non-blood liquid biopsy matrices may expand the temporal biomarker toolkit [61]. If validated prospectively, temporal RNA signatures could function as early indicators of transcriptional adaptation, identifying emerging resistance states before radiologic progression or clinical deterioration becomes evident. The clinical value of temporal RNA biomarkers may lie not in detecting disease presence but in anticipating disease trajectory. The integration of spatial and temporal RNA dimensions is illustrated in Figure 3.

## 6. Deployable RNA Assays

For RNA-based biomarkers to transition from research discovery to clinical impact, they must meet a set of analytical and clinical requirements that go beyond statistical association with outcomes. A deployable RNA biomarker requires five elements: analytical reproducibility across technical replicates and platforms, a defined cutoff strategy that generates binary or tiered clinical decisions, stability across the pre-analytical and analytical variables encountered in clinical workflows, a clear decision consequence that connects the biomarker result to a specific management action, and validation across independent cohorts of sufficient size and clinical representativeness [46].

Tissue-based RNA panels may represent the most mature format for near-term clinical deployment. Jiang et al. (2016) developed an integrated mRNA-lncRNA signature in TNBC with predictive and prognostic value, providing one of the earliest demonstrations that multi-RNA panels can outperform single-gene markers and genomic signatures in TNBC risk stratification [62]. Supplitt et al. (2025) identified RNA-based predictive biomarkers for chemotherapy sensitivity in TNBC using transcriptomic analysis, illustrating the continued potential of tissue-based RNA profiling for treatment selection [63]. Tissue-based panels satisfy several deployment criteria: FFPE-compatible extraction methods are established, multi-gene expression assays have regulatory precedents in other cancer types (such as Oncotype DX in hormone receptor-positive breast cancer), and defined histological platforms allow spatial co-registration with other pathological information.

Liquid biopsy RNA assays may present a more complex deployment landscape but offer the temporal sampling capability that tissue biopsies cannot provide. EV RNA panels satisfy the analytical requirement of measurability in plasma with defined pre-analytical protocols, though inter-laboratory variability in EV isolation remains a challenge. The standardization of vesicle isolation methods is a prerequisite to multi-site RNA biomarker validation, and the choice of isolation protocol substantially affects the detected RNA profile [60,64]. Das et al. (2023) reviewed EV RNA in TNBC with respect to immune regulation and biomarker potential and highlighted that the biological relevance of EV cargo, including miRNA, lncRNA, and circRNA species, is increasingly supported by mechanistic studies, but that clinical validation studies with prospective design and pre-specified endpoints are needed [65]. EV RNA also satisfies the stability criterion in principle, as the vesicle membrane protects RNA cargo from plasma RNase activity, but storage and freeze–thaw conditions require careful standardization.

Wen, Tang, and Zou (2025) demonstrated a dual-function RNA biomarker approach in TNBC that integrates relapse prediction with immune profiling, providing an example of an RNA signature that meets the “clear decision consequence” criterion by linking classifier output directly to immune context and potential immunotherapy candidacy [45]. Beňačka et al. (2024) reviewed non-coding RNAs as biomarkers in breast cancer with attention to diagnostic implications, documenting a range of validated ncRNA candidates with reproducible measurement properties [66]. The Zhu, Wang, and Xu (2025) prognostic model based on the ceRNA network in TNBC illustrates how multi-RNA integrative signatures, incorporating lncRNA, miRNA, and mRNA interaction networks, can be distilled into deployable prognostic models, provided that the component biomarkers are individually validated for their analytical performance [67].

## 7. Toward a State-Guided Adaptive Management Framework for TNBC

### 7.1. The Conceptual Shift

TNBC is not a disease with a fixed molecular identity but a system that evolves under selective pressure, therapeutic exposure, and microenvironmental influence. The current paradigm of biomarker-guided TNBC management is predominantly baseline-oriented: patients are classified at diagnosis based on PD-L1 status, tumor-infiltrating lymphocyte density, or germline BRCA mutation status, assigned to an initial treatment regimen, and subsequently monitored by radiologic imaging until progression is declared. This paradigm treats the tumor as a static object with fixed properties and updates the clinical strategy only when macroscopic failure is evident. Given the evidence reviewed above that transcriptional programs shift under therapy, that resistance emerges through regulatory reprogramming rather than solely through clonal selection of pre-existing variants, and that the spatial and temporal distribution of cell states contains clinically actionable information that single-timepoint measurements miss, it is reasonable to conclude that this static paradigm is insufficient.

Herein, a state-guided adaptive framework is proposed in which RNA signatures function as dynamic measurement layers informing therapy selection, monitoring, and modification across disease evolution. The operating logic of this framework is iterative: measure the current transcriptional state, select therapy informed by that state, monitor for state transitions during therapy, detect early evidence of adaptive reprogramming, and modify therapy in response to state change before radiologic progression. This “measure–adapt–re-measure” cycle represents a fundamentally different architecture for oncology decision making, one that treats the biomarker not as a one-time classifier but as a continuous readout of a moving biological system. Han et al. (2025) articulated the conceptual basis for this approach in the context of single-cell transcriptomics and metastatic breast cancer, framing tumor evolution and therapeutic resistance as processes that require longitudinal molecular tracking rather than static profiling [42].

### 7.2. The Adaptive Loop: Core Architecture

The adaptive management cycle consists of five steps that together define the operational structure of the proposed framework.

Step 1: Baseline State Profiling: At diagnosis or prior to treatment initiation, a tissue RNA panel is applied to classify the tumor into functionally meaningful states: immune-active, immune-excluded, stress-adaptive, or stem-like/persister-prone. This classification is informed by lncRNA and mRNA expression profiles that have been validated as correlates of these states in TNBC cohorts [45,62]. Critically, RNA classification complements rather than replaces genomic profiling, germline and somatic DNA data continue to inform mutation-driven treatment decisions, and RNA state profiling adds the layer of current transcriptional program and transition propensity. The clinical question at Step 1 is as follows: which therapy should be initiated, and what is the baseline state against which subsequent changes will be measured?

Step 2: On-Therapy Monitoring: During treatment, serial ctRNA and EV RNA measurements are performed at defined intervals, ideally at cycle boundaries or after defined cumulative dose thresholds, to detect transcriptional shifts before radiologic progression. Sadovska et al. (2022) demonstrated that EV RNA profiles change measurably during neoadjuvant chemotherapy in breast cancer [43], and Syeda et al. (2025) documented immune monitoring dynamics during neoadjuvant chemo-immunotherapy in TNBC [5], establishing that immune transcriptional programs evolve in real time during treatment. The clinical question at Step 2 is the following: is the tumor transitioning toward a resistant transcriptional state, and, if so, at what rate?

Step 3: Early Resistance Detection: When on-therapy RNA monitoring detects the emergence of drug-tolerant persister programs, immune evasion signatures, or alternative survival pathway activation, this triggers a clinical decision point before radiologic progression is documented. Baudre et al. (2026) identified a shared persistence program in TNBC that could serve as a molecular target for early resistance detection [19]. The clinical question at Step 3 is as follows: should therapy be escalated, modified, or switched to prevent phenotypic resistance from becoming entrenched?

Step 4: Spatial Reassessment in Resistant Disease: When resistance is suspected or confirmed, spatial transcriptomic reassessment of a new biopsy enables identification of the spatial architecture of resistance—whether resistance is distributed uniformly across the tumor or concentrated in anatomically defined niches. Bassiouni et al. (2023) and Wang et al. (2024) demonstrated that resistant spatial niches in TNBC have distinct RNA expression profiles that can be identified by spatial transcriptomics [49,52], and Kim et al. (2024) showed that residual disease after chemo-immunotherapy has a characteristic spatial transcriptional program [56]. Liu et al. (2024) demonstrated in glioblastoma that spatial transcriptomics can reveal the segregation of tumor cell states and identify immunosuppressive niches, a principle directly applicable to TNBC spatial resistance mapping [68]. The clinical question at Step 4 is as follows: is resistance uniform and therefore a systemic treatment problem, or spatially restricted and potentially addressable by local or combination strategies?

Step 5: Iterative Adaptation: The measure–adapt–re-measure cycle is explicitly recursive. Following any therapeutic modification triggered by RNA-informed decision points, a new monitoring baseline is established and the cycle repeats. This iterative adaptation is the “operating system” of the proposed framework: it does not seek to predict the entire trajectory of the tumor upfront but rather to continuously update the clinical strategy in response to measured biological changes. Ultimescu et al. (2026) provided empirical support for the necessity of this approach by documenting that molecular subtypes shift during and after treatment in ways that cannot be predicted from baseline profiling [3].

The clinical application of state-aware RNA biomarkers requires a structured translational pathway that links measurement modalities to biological interpretation and clinical decision making. Table 3 outlines a stage-specific roadmap integrating tissue-based and liquid biopsy RNA measurements across the TNBC disease continuum, highlighting how RNA-based readouts may inform adaptive clinical strategies. The operational structure of this adaptive loop, derived from the state-aware RNA architecture, is illustrated above in Figure 2.

To illustrate the potential clinical application of this framework, we present a hypothetical scenario in Box 1.

Box 1 **Hypothetical clinical scenario: RNA-guided adaptive management in TNBC.**

  This scenario represents a conceptual, investigational framework and does not reflect current standard-of-care practice.

- A 48-year-old patient presents with newly diagnosed stage IIIC TNBC. Standard diagnostic evaluation is performed, including histopathological assessment and genomic profiling. In parallel, the patient is enrolled in a prospective translational protocol incorporating tissue-based RNA profiling.
- At baseline, integrated mRNA and ncRNA signatures classify the tumor within an immune-excluded transcriptional state, accompanied by molecular features suggestive of elevated transition potential toward adaptive resistance programs. This classification is not used as a standalone clinical determinant but rather as an additional layer of biological context, complementing established clinicopathologic and genomic parameters.
- Based on current clinical guidelines, neoadjuvant chemo-immunotherapy is initiated. Within the investigational framework, serial liquid biopsies are collected during treatment to assess ctRNA and EV-associated RNA. These measurements are performed for exploratory purposes, aiming to capture temporal changes in transcriptional programs.
- Following two treatment cycles, RNA profiling reveals a shift in ncRNA-associated regulatory patterns consistent with transcriptional programs previously linked to drug-tolerant persister (DTP) states. These findings are interpreted within the context of emerging biological evidence and are not considered independently sufficient to guide treatment modification outside of the study protocol.
- Within the predefined structure of the translational study, such molecular signals may trigger protocol-specified reassessment, including multidisciplinary review and, where appropriate, consideration of therapeutic adjustment strategies targeting pathways implicated in adaptive resistance. These decisions are made in conjunction with clinical, radiologic, and pathological evaluation, rather than replacing them.

### 7.3. Standards for Clinical Translation

For the proposed framework to move from concept to clinical practice, defined analytical and clinical validity standards must be met at each step. Analytical validity requires that the RNA biomarkers used in each step of the adaptive loop demonstrate reproducibility across technical replicates, cross-platform consistency between sequencing-based and hybridization-based measurement platforms, and pre-analytical stability across the specimen processing conditions encountered in multi-site clinical trials. Clinical validity requires that each RNA signature used to inform a decision point is prospectively associated with the clinical outcome it is intended to predict, response to immunotherapy, emergence of resistance, and MRD status, with pre-specified performance thresholds including sensitivity, specificity, and positive predictive value in representative TNBC populations.

Clinical utility, the highest standard, requires demonstration that clinical decisions guided by RNA biomarker results improve patient outcomes compared to standard management. This standard has not yet been met for RNA-based adaptive strategies in TNBC, and its pursuit requires prospective interventional trials specifically designed to test the decision–consequence logic of the framework. Threshold definition is a particularly critical requirement: what level of RNA shift in serial liquid biopsy measurements constitutes a clinically meaningful transition, and what is the acceptable window between detection and therapeutic intervention? Without pre-specified thresholds, RNA-guided adaptation risks becoming subjective and unstandardized, replicating the inconsistency that currently plagues static biomarker interpretation.

Clinical translation of RNA-based biomarker strategies will require prospective validation studies demonstrating that RNA-defined state transitions precede clinically actionable events. Trial designs may include adaptive monitoring frameworks in which serial RNA profiling is used to detect emerging resistance programs and trigger therapy modification. Such approaches would test whether RNA-guided intervention improves outcomes compared with static baseline biomarker selection alone.

### 7.4. Trial Design Implications

The adaptive management framework implies a set of trial design innovations that move beyond conventional randomized controlled trial structures. Baseline RNA-stratified arms, in which patients are randomized within RNA-defined state categories (immune-active, immune-excluded, persister-prone) rather than by standard clinical features alone, would allow the interrogation of state-specific treatment effects. On-treatment RNA-triggered switch arms, in which the detection of a pre-specified RNA signature during therapy triggers a protocol-defined treatment modification, would provide the first prospective evidence for the clinical utility of RNA-based adaptive management. RNA-guided escalation strategies, in which the intensification of immunotherapy, addition of ADC therapy, or introduction of epigenetic reprogramming agents is triggered by RNA signatures of immune evasion or drug tolerance, represent a more complex but potentially highly impactful design. MRD-guided RNA surveillance studies, in which serial EV RNA profiling is used alongside ctDNA in post-treatment surveillance, would address the sensitivity gap in current MRD detection and test whether RNA-based MRD signals predict relapse independently of ctDNA. These designs do not require fully validated biomarkers at their outset; the trials themselves would generate the validation data, but they do require pre-specified decision rules, defined RNA measurement timepoints, and outcome assessments aligned with the hypothesized biology.

### 7.5. Limitations and Practical Barriers

The framework proposed here faces significant practical barriers that must be acknowledged. RNA instability is a fundamental concern in clinical specimen handling: mRNA and lncRNA are substantially more labile than DNA, and the quality of RNA extracted from FFPE tissues or liquid biopsy samples depends critically on pre-analytical conditions including time from collection to processing, fixation duration for tissue, and freeze–thaw cycles for plasma. Standardization across clinical sites is therefore not merely desirable but prerequisite, and multi-site studies must include rigorous pre-analytical quality control with validated metrics.

The standardization of RNA measurement platforms presents a second challenge. Unlike DNA sequencing, which has converged on relatively standardized workflows, RNA profiling can be performed by bulk RNA-seq, targeted panels, hybridization capture, digital counting (NanoString), or qRT-PCR, and the results of these methods are not directly interchangeable. A deployable RNA biomarker must define the measurement platform as part of the assay specification and validate performance separately on each platform intended for clinical use.

Liquid biopsy RNA approaches face sensitivity limits in early-stage disease and in patients with low disease burden, where EV RNA signal may be too low for reliable quantification. The integration of RNA liquid biopsy with orthogonal modalities, ctDNA, circulating tumor cells, and protein biomarkers is likely necessary to achieve clinically adequate sensitivity across the full range of TNBC disease states and treatment contexts.

Although numerous studies have linked ncRNAs to therapy resistance, EMT, immune modulation, stemness, and stress adaptation, many of these associations remain correlative rather than mechanistically resolved [15,66]. Reviews of lncRNAs and circRNAs in breast cancer emphasize that ncRNAs are frequently embedded within highly interconnected and context-dependent regulatory networks, making it difficult to distinguish whether a given transcript functions as a driver of phenotypic transition, a stabilizer of adaptive states, or simply a byproduct of broader transcriptional reprogramming [9,13].

Tumor heterogeneity within biopsies is another limitation. Even with spatially resolved methods, a single biopsy sample represents only the accessible portion of a potentially heterogeneous tumor, and the spatial architecture of RNA-defined states may differ between the biopsy site and the clinically dominant tumor compartment.

Clinically meaningful correlations between ncRNA trajectories and long-term outcomes such as relapse, resistance evolution, and survival will require prolonged prospective follow-up in large patient cohorts. Given the relatively long timelines required for recurrence assessment and therapeutic outcome maturation in breast cancer, the development of sufficiently validated longitudinal datasets capable of defining clinically actionable thresholds will likely require more than a decade. Future studies must move beyond exploratory association analyses and instead address clearly defined clinical questions regarding how RNA-guided interventions should influence patient management. Dynamic immune monitoring strategies must be meaningfully integrated into therapeutic decision-making frameworks rather than functioning solely as observational biomarkers [5]. Similarly, for ctDNA-based minimal residual disease detection, critical uncertainties remain regarding optimal testing intervals, intervention thresholds, and appropriate therapeutic responses following biomarker detection [10,11].

## 8. Conclusions

TNBC presents a biomarker challenge that static, genomic-centric strategies are structurally ill-equipped to address. The dynamic transcriptional biology of the disease, its plasticity under therapy, its spatially heterogeneous immune architecture, and its capacity to generate drug-tolerant persister populations through non-genetic reprogramming demand measurement strategies that are themselves dynamic, spatially resolved, and sensitive to the regulatory logic of cell state transitions. RNA, and particularly ncRNA, may provide a biologically appropriate substrate for this kind of measurement: it reflects active transcriptional programs and, uniquely, encodes the regulatory threshold information that determines whether a tumor will transition into a resistant state. A state-guided adaptive management framework, in which RNA signatures are used as iteratively updated measurement layers informing therapy selection and modification, may represent the logical clinical translation of this biology. Realizing this framework requires the field to advance analytical standards, define clinical validity criteria, and design prospective trials in which RNA-guided decisions are tested against outcome endpoints. The conceptual foundation is sound, the biological rationale is compelling, and the clinical need is urgent—what remains is translation. This framework can shift oncology from classification-based medicine to state-tracking medicine. The challenge in TNBC biomarker development is no longer discovery but translation from static molecular description to dynamic, state-aware measurement systems capable of guiding adaptive clinical decision making.

## Figures and Tables

**Table 1:** Static limitations and potential dynamic solutions.

Biomarker/Theme	Static Limitation & Evidence	Dynamic/Emerging Insight & Evidence
PD-L1 Expression (IHC)	Static PD-L1 IHC shows technical and biological heterogeneity across assays and within tumors; single biopsy may not capture the current immune state, undermining predictive utility in TNBC immunotherapy selection [5].	Spatial and temporal profiling of PD-L1 with immune context (e.g., TILs, immune gene signatures); repeated sampling may better reflect treatment-induced changes in tumor–immune dynamics [5].
Tumor Mutational Burden (TMB)	Review states “promise”; lack of standardization and cutoffs plus disease heterogeneity limit clinical predictivity of static TMB measurements for ICI response in TNBC [7].	Future directions: integrating TMB with other biomarkers and multi-omic data longitudinally to improve prediction of ICI benefit and capture evolving tumor states [7].
ctDNA for MRD & Monitoring	Static, single-timepoint MRD detection (e.g., one post-treatment ctDNA draw) can miss tumor evolution; sensitivity issues remain a challenge [11].	Longitudinal monitoring across neoadjuvant, post-neoadjuvant, and adjuvant timepoints reflects tumor dynamics, predicts pathological response and relapse risk, and may outperform static measures alone [10,11].
General TNBC Heterogeneity	Static single-site biopsies (PD-L1, genomic markers) do not capture spatial and temporal tumor heterogeneity, leading to misclassification or poor treatment stratification [5].	Dynamic, longitudinal profiling is needed to capture evolving tumor biology and response/resistance mechanisms: repeated biopsies, ctDNA/CTC tracking, and multi-modal assessments. Radiomic and immune monitoring approaches are being developed to augment static assessments [5].

**Table 2:** Non-coding RNAs Organized by Biological State Domains in TNBC.

Biological State Domain	Representative ncRNAs	Proposed Biomarker Potential	State-Aware Relevance
Immune Exclusion & Immune Architecture	USP30-AS1 (exosomal lncRNA)	Associated with immune response in TNBC; example from immune-related lncRNA pair model identifying prognostic markers [18]	Reflects immune activation vs. immune suppression states; correlates with PD-L1 expression and tumor–immune interaction dynamics
	LINC01614 (lncRNA)	Poor prognostic marker in breast cancer [17]	Regulated by TGF-β and FAK signaling; encodes stromal remodeling and immune exclusion programs
	miR-155	Prognostic marker; linked to immune response and chemotherapy sensitivity [24,25]	Reflects immune-enriched and inflammatory tumor states
Epithelial–Mesenchymal Transition (EMT) & Plasticity	miR-200 family (miR-200a/b/c)	Prognostic marker associated with metastatic risk [26,27]	Canonical regulators of EMT; loss indicates transition toward mesenchymal and invasive states
	circANKS1B	Prognostic marker linked to metastasis [28]	Promotes TGF-β-driven EMT; reflects transition toward metastatic competence
	HOTAIR	Associated with metastasis and poor survival [29,30]	Marker of chromatin reprogramming and EMT-associated transition stability
Drug-Tolerant Persistence & Therapy Adaptation	MALAT1	Associated with recurrence and metastasis [31,32]	Linked to alternative splicing, EMT plasticity, and survival signaling in adaptive states
	NEAT1	Associated with chemoresistance and hypoxia response [33,40]	Reflects stress-adapted and therapy-resistant tumor states
	LINC00511	Prognostic marker associated with poor overall survival [34]	Linked to proliferative and invasive transition programs
Stress Response & Proliferative Adaptation	miR-21	Associated with poor outcomes; potential treatment response marker [35,36]	Reflects anti-apoptotic and proliferative stress-adaptation states
	miR-34a	Tumor suppressor miRNA; prognostic marker [37,38]	Reflects p53 functional integrity, stemness control, and EMT balance
	miR-221/222	Associated with aggressive phenotype and poor prognosis [39]	Reflect proliferative and therapy-resistant transition states

**Table 3:** Translational roadmap for RNA-based state-aware biomarkers in TNBC.

Clinical Stage	Measurement Modality	RNA-Based Readout	Biological Insight	Clinical Decision Impact	Translational Status
Baseline Diagnosis	Tissue RNA panel (mRNA + ncRNA)	State classification (immune-active, immune-excluded, persister-prone)	Defines current transcriptional state and transition potential	Guides initial therapy selection (e.g., IO suitability, combination strategies)	Early clinical application (retrospective/prognostic models)
Early On-Treatment	Liquid biopsy (ctRNA, EV RNA)	Dynamic RNA shifts (ncRNA-driven transition signals)	Detects early adaptive reprogramming under therapy	Identifies emerging resistance before radiologic progression	Emerging (proof-of-concept studies)
Mid-Treatment Monitoring	Serial RNA profiling	Persistence signatures, EMT activation, immune suppression programs	Tracks trajectory of tumor evolution and therapy response	Supports continuation vs. early therapy modification	Experimental (requires prospective validation)
Early Resistance Detection	Longitudinal RNA trend analysis	Drug-tolerant persister (DTP) signatures, stress-response ncRNAs	Identifies transition into resistant states prior to phenotypic manifestation	Enables early intervention (switch/escalation/combination therapy)	Conceptual to early translational
Progression Assessment	Tissue + spatial transcriptomics	Spatial RNA heterogeneity (resistant niches, immune architecture)	Map location and structure of resistance within tumor microenvironment	Differentiates systemic vs. localized resistance → informs therapy strategy	Emerging (limited clinical integration)
Post-Treatment Surveillance (MRD)	Liquid biopsy (ctRNA ± ctDNA integration)	Low-level RNA signals indicating residual adaptive programs	Detects subclinical disease and transcriptional reactivation	Predicts relapse risk and informs early intervention	Early-stage research
Integrated Clinical Decision System	Multi-timepoint RNA framework	Combined spatial + temporal RNA			

## Data Availability

No new data were created or analyzed in this study. Data sharing is not applicable to this article.
